# Composition, Formation, and Regulation of the Cytosolic C-ring, a Dynamic Component of the Type III Secretion Injectisome

**DOI:** 10.1371/journal.pbio.1002039

**Published:** 2015-01-15

**Authors:** Andreas Diepold, Mikhail Kudryashev, Nicolas J. Delalez, Richard M. Berry, Judith P. Armitage

**Affiliations:** 1 Department of Biochemistry, University of Oxford, Oxford, United Kingdom; 2 Biozentrum, University of Basel, Basel, Switzerland; 3 Clarendon Laboratory, Department of Physics, University of Oxford, Oxford, United Kingdom; Rutgers University-Robert Wood Johnson Medical School, UNITED STATES

## Abstract

Many gram-negative pathogens employ a type III secretion injectisome to translocate effector proteins into eukaryotic host cells. While the structure of the distal “needle complex” is well documented, the composition and role of the functionally important cytosolic complex remain less well understood. Using functional fluorescent fusions, we found that the C-ring, an essential and conserved cytosolic component of the system, is composed of ~22 copies of SctQ (YscQ in *Yersinia enterocolitica*), which require the presence of YscQ_C_, the product of an internal translation initiation site in *yscQ*, for their cooperative assembly. Photoactivated localization microscopy (PALM) reveals that *in vivo*, YscQ is present in both a free-moving cytosolic and a stable injectisome-bound state. Notably, fluorescence recovery after photobleaching (FRAP) shows that YscQ exchanges between the injectisome and the cytosol, with a t_½_ of 68 ± 8 seconds when injectisomes are secreting. In contrast, the secretin SctC (YscC) and the major export apparatus component SctV (YscV) display minimal exchange. Under non-secreting conditions, the exchange rate of YscQ is reduced to t_½_ = 134 ± 16 seconds, revealing a correlation between C-ring exchange and injectisome activity, which indicates a possible role for C-ring stability in regulation of type III secretion. The stabilization of the C-ring depends on the presence of the functional ATPase SctN (YscN). These data provide new insights into the formation and composition of the injectisome and present a novel aspect of type III secretion, the exchange of C-ring subunits, which is regulated with respect to secretion.

## Abstract 2

The injectisome is a membrane complex through which some bacteria can inject effector proteins into host cells. This study reveals that the cytosolic C-ring structure has a dynamic relationship to the rest of the injectisome, with implications for the regulation of secretion.

## Author Summary

The type III secretion system, also known as the injectisome, is a key virulence factor in many gram-negative bacteria, and is responsible for the transmission of bacterial proteins directly into host cells. While some elements of the system are well characterized, the cytosolic components involved in substrate recognition and handling are not well understood. One of the major questions is the role of the C-ring, an essential yet enigmatic cytosolic injectisome member. We used fluorescence microscopy to analyze the architecture and behavior of the C-ring in live *Y. enterocolitica* bacteria, a human pathogen. We found that *in vivo*, the C-ring assembles cooperatively with the help of additional copies of its own C-terminal fragment and has a highly dynamic structure, with C-ring subunits exchanging between the working injectisomes and a cytosolic pool. The rate of exchange is different between secreting and non-secreting injectisomes and depends on the function of the type III secretion ATPase, indicating that the stability of the complex is altered when functioning. This dynamic behaviour raises the possibility that the C-ring is a regulator of targeted protein delivery by the type III secretion system and makes the C-ring a viable target for the development of novel anti-virulence drugs.

## Introduction

Bacteria that live in close contact with eukaryotic cells are frequently able to modulate host cell behavior. Various Gram-negative species employ a molecular syringe, the type III secretion system (T3SS) or injectisome [1, 2], to translocate effector proteins from the bacterial cytosol into the host cell. T3SSs are often involved in pathogenesis, and are crucial virulence factors for animal and plant pathogens [3–5], but are also employed to promote symbiosis [6, 7]. Thus, the translocated effectors vary greatly between species [8, 9]. In contrast, the export machinery itself is conserved across organisms [10, 11]. It is a large complex that comprises more than 15 different proteins present in one to more than a hundred copies each (S1 Fig). The distal part of the injectisome including the needle and the “base,” a series of membrane-spanning rings, is structurally well characterized [12, 13]. However, little is known about the structure and function of the proximal components, the “export apparatus” in the inner membrane (IM) and the cytosolic components that are essential for the function and regulation of the system. This report uses the unified Sct protein nomenclature [3] for general protein features, in combination with the *Yersinia* Ysc nomenclature (S1 Fig; S1 Table for names of homologous proteins).

Five conserved cytosolic proteins are essential for type III secretion, an ATPase (SctN; YscN in *Yersinia)*, thought to detach chaperones and unfold export substrates [14], and its putative negative regulator (SctL; YscL) [15, 16]; a protein with homology to the central stalk of the FoF1-ATPase that stimulates ATPase activity (SctO; YscO) [17–19]; a homolog of the flagellar motor C-ring components FliM and FliN (SctQ; YscQ) [20]; and an additional accessory protein (SctK; YscK). All of these proteins interact with each other: yeast-two-hybrid and a yeast-three-hybrid experiment clearly suggest a sequence of interactions SctK-SctQ-SctL-SctN [20–23] while SctO has been shown to directly and functionally interact with SctN and SctL (S1 Fig) [19, 24]. The ATPase SctN and the C-ring component SctQ require each other and the two interacting proteins SctK and SctL to assemble at the cytosolic interface of the injectisome [25], suggesting the formation of one large cytosolic complex. The cytosolic components are required for various essential steps in type III secretion, including energy transduction, binding (or rejection) of substrates, and their preparation for export [14, 18, 23, 26, 27]. Despite this central role, surprisingly little is known about the molecular events and the organization of the proteins at the cytosolic interface, mostly because the components rarely co-purify with the rest of the machinery and are difficult to analyze *in vitro* in the absence of the other parts of the injectisome.

The T3SS is related to the bacterial flagellar motor with which it shares a common ancestor (see S1 Table for homologues) [28–31]. Recently, this homology was highlighted by the discovery that at least in some organisms, the cytosolic C-ring, which is formed by the two proteins FliM and FliN in the flagellum, also consists of two different proteins arising from a single gene in the injectisome [32, 33]: (i) the full length protein, SctQ_full_, which is similar in size and has some sequence homology to FliM in its N-terminal region, and (ii) a product from an internal translation start site, SctQ_C_, which comprises about the C-terminal third of SctQ and is highly homologous to FliN. The flagellar C-ring is part of the switch complex [34–36], which can reverse the direction of rotation of the *Escherichia coli* flagellum upon binding of the activated response regulator CheY-P to FliM, allowing the bacterium to tumble and reorient [37, 38]. The C-ring is also required for the export of substrates through the flagellar T3SS, but this can be overcome by simple overexpression of the transcriptional regulators [39] or even the ATPase alone [40].

The injectisome is generally not thought to rotate. Remarkably however, the C-ring not only remains conserved among all known injectisomes, but is also absolutely essential for export of substrates, suggesting a divergent functional adaptation in the flagellum and the injectisome. This idea is supported by recent cryo-tomography studies [41, 42], where the C-ring, a prominent feature of the flagellum [42, 43], was absent in the injectisome, whereas both the major export apparatus component (FlhA in the flagellum/SctV in the injectisome) and the ATPase (FliI/SctN) formed distinct and comparable structures in both machines. Consequently, the structure and composition of the injectisome C-ring remain unclear. Similarly, its localization is ambiguous. The C-ring protein SctQ is present at the cytosolic interface of the injectisome [20] and co-localizes with other injectisome components [25]. However, complexes of the cytosolic components including SctQ only partially cofractionated with other injectisome components and were also found in the cytosolic fraction [21, 26], indicating that the C-ring is either not tightly bound or is present in two subpopulations. Insights into the role of the injectisome C-ring have been gained by interaction studies showing that the C-ring is involved in export cargo handling [23, 26]. In combination with SctK and SctL, the C-ring was shown to form a “sorting platform” that governs the order of substrate export [26]. However, the molecular mechanism of this function remains as elusive as structure and localization of the C-ring.

To study the composition and functional role of the injectisome C-ring, we generated and studied functional fluorescent fusions of various injectisome components in a *Y. enterocolitica* strain lacking the major virulence effectors [41]. Based on relative fluorescence intensities, we found that there are 22 ± 8 full-length C-ring subunits YscQ per injectisome. YscQ_C_, the product of the internal translation start site, is not only required for substrate translocation, but also necessary for the localization of YscQ_full_ and the correct assembly of the ATPase. YscQ exchanges between its docking site at the injectisome and a cytosolic pool *in vivo*. Its exchange rate under secreting conditions is significantly higher than under non-secreting conditions (t_½_ of 68.2 ± 7.9 s versus 134.3 ± 16.1 s), which links C-ring dynamics and effector export. This correlation depends on an active ATPase, suggesting a close functional relation between the two largest cytosolic components of the injectisome, C-ring and ATPase.

## Results

### The C-ring Comprises about 22 Subunits of YscQ

**Figure 1:**
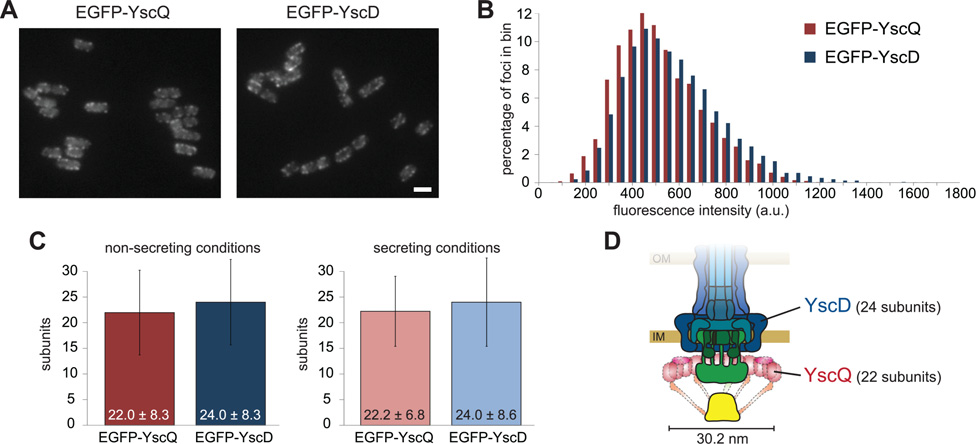
The C-ring comprises approximately 22 YscQ subunits per injectisome. (A) Fluorescence micrographs of bacteria expressing EGFP-YscQ (left) or EGFP-YscD (right) from their native genetic environment under non-secreting conditions. Scale bar, 2 μm. (B) Intensity distribution of analyzed foci under non-secreting conditions (*n* > 3,000). (C) Relative stoichiometry of YscQ under non-secreting (left) and secreting (right) conditions, using YscD = 24 as a reference. Error bars represent standard deviations. (D) Schematic representation of an injectisome basal body with a C-ring diameter of 30.2 nm (see S1 Text; representation approximately to scale, based on [45]). Note that conformation and localization of the dashed components, including YscQ, have not been experimentally determined. Numerical values and raw data for (B) and (C) can be found in S1 Data.

The composition of the C-ring is unknown. To estimate the number of YscQ proteins within the *Yersinia* T3SS, we compared the relative intensities of foci in bacteria expressing either EGFP-YscQ or EGFP-YscD. Members of the SctD family, to which YscD belongs, have been shown to form 24-mer ring structures [12, 44–48], which was recently corroborated for *Yersinia* by the crystal structure of YscD and the cryotomography structure of the injectisome [41]. We therefore devised a spot-detection algorithm and used the intensity of EGFP-YscD foci as a reference for EGFP-YscQ. Both proteins were expressed from the pYV virulence plasmid using their native promoters. For biosafety reasons, this and all subsequent experiments were performed in *Y. enterocolitica* strains that lack the major virulence effectors YopH, O, P, E, M, T and are auxotrophic for diaminopimelic acid [41]. While EGFP-YscQ was fully functional, EGFP-YscD was slightly less efficient in effector export. Importantly, both fusion proteins were stable, as detected by an immunoblot of total cellular proteins, using anti-GFP antibodies (S2 Fig). Based on the relative intensities of the foci, we calculated the number of YscQ_full_ per injectisome to be 22.0 ± 8.3 under non-secreting conditions and 22.2 ± 6.8 under secreting conditions (Fig. 1). As the intensity of foci is determined at the microscopy plane corresponding to the center of the bacterium in the respective DIC image, some foci will be centered below or above this plane and will be detected with lower intensity, accounting for most of the observed variance. Supporting this, the intensity distribution curve for YscQ did not show any greater width than the curve for YscD (Fig. 1B and 1C). As the number of YscD per injectisome is thought to be fixed, this suggests that the stoichiometry of YscQ at the injectisome is similarly constant.

### The Short Variant of YscQ Is Required for the Assembly of the Cytosolic Complex of the Injectisome

**Figure 2:**
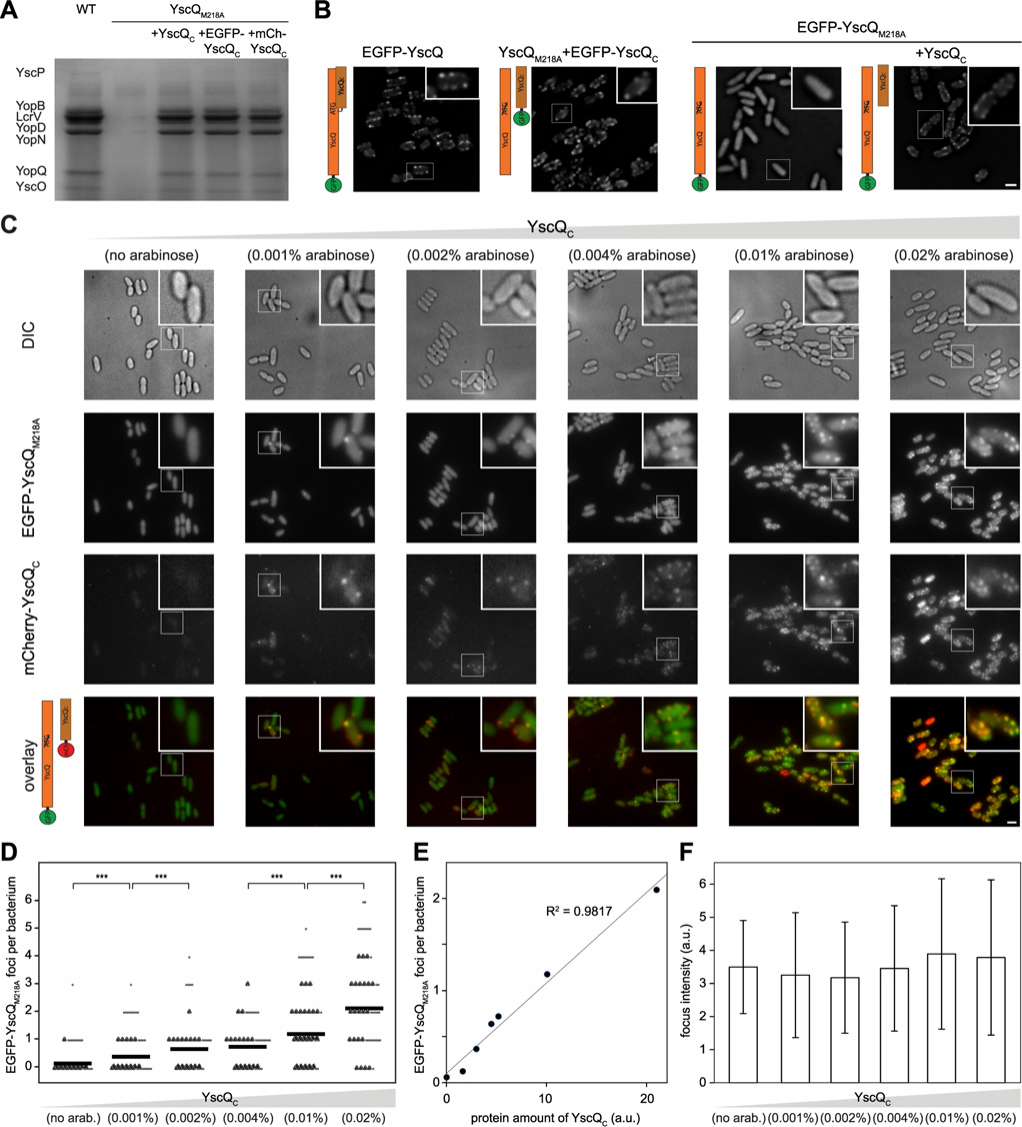
YscQ_C_, the product from an internal translation initiation site in *yscQ*, is required for the formation of C-ring foci, which is cooperative. Strains expressing YscQ_M218A_ from the native promoter and therefore lacking YscQ_C_ do not secrete effectors and YscQ_M218A_ requires YscQ_C_ for localization at the injectisome. YscQ_C_ and YscQ_M218A_ colocalize; increased expression levels of mCherry-YscQ_C_ lead to an increase in spot number, but not spot intensity for EGFP-YscQ_M218A_. (A) Secretion assay showing the secreted proteins in a wild-type (WT) strain, and YscQ_M218A_, uncomplemented or complemented *in trans* with YscQ_C_, EGFP-YscQ_C_, or mCherry-YscQ_C_. (B) Fluorescence micrographs showing the distribution of EGFP-YscQ, EGFP-YscQ_C_, and EGFP-YscQ_M218A_, uncomplemented or complemented by YscQ_C_ *in trans*. (C) Cellular distribution of EGFP-YscQ_M218A_ (expressed from its native promoter on the virulence plasmid, second row) and mCherry-YscQ_C_ (expressed in increasing amounts *in trans* induced by the given concentrations of arabinose, third row). The overlay (bottom row) displays the colocalization of both YscQ versions. Scale bars, 2 μm. (D) Number of detected EGFP-YscQ_M218A_ foci per bacterium in cells expressing increasing amounts of mCherry-YscQ_C_ (as in (C)). *n* > 170 cells per condition (see Materials and Methods for details). Black lines represent the average number of foci per bacterium; circles represent the number of foci per single bacterium (arranged in groups of ten). ***, *p* < 0.001. (E) Average number of detected EGFP-YscQ_M218A_ foci per bacterium in relation to the amount of mCherry-YscQ_C_, as quantified on an immunoblot with a polyclonal anti-YscQ antibody (S4 Fig). Data points from left to right: no YscQ_C_ (no plasmid), and increasing amounts of mCherry-YscQ_C_ (no arabinose, 0.001%, 0.002%, 0.004%, 0.01%, 0.02% arabinose, as in (C)). (F) Average intensity of foci for the bacteria analyzed in (D)). Error bars represent standard deviations of all foci. Numerical values and raw data for (D–F) can be found in S1 Data.

It was recently found that an internal translation initiation site in SctQ leads to the expression of a short C-terminal fragment, SctQ_C_, which interacts with SctQ_full_ [32, 33]. We deleted the internal start site of the *Yersinia* C-ring protein YscQ by mutating the corresponding Met218 to Ala and found that the resulting strain lacking YscQ_C_ did not export any effectors or translocators, confirming that the additional C-terminal fragment is essential for secretion in *Yersinia* [33]. This effect could be complemented *in trans* by YscQ_C_, EGFP-YscQ_C_, or mCherry-YscQ_C_ (Fig. 2A). EGFP-YscQ_C_ was stable (S2 Fig) and localized in foci at the bacterial membrane (Fig. 2B), like other fluorescent injectisome components [25, 49]. Surprisingly, when we analyzed the distribution of EGFP-YscQ_M218A_, the full-length protein lacking the internal start site, it no longer localized to the injectisome, but was completely cytosolic. Upon *in trans* expression of YscQ_C_, localization was recovered (Fig. 2B). Therefore, although YscQ_full_ contains all parts of YscQ_C_, it requires additional YscQ_C_ for proper localization. Similarly, EGFP-YscQ_C_ did not localize in foci in the absence of YscQ_full_ (S3A Fig), showing that only the YscQ_full_:YscQ_C_ complex can correctly assemble. To test whether localization of the ATPase YscN also depends on YscQ_C_, we imaged EGFP-YscN in YscQ_M218A_. In the resulting absence of YscQ_C_, EGFP-YscN remained completely cytosolic (S3B Fig), indicating that YscQ_C_ is required for the assembly of the complete cytosolic complex.

### Colocalization and Cooperative Binding of the C-ring Subunits

The expression of YscQ_C_ from plasmid revealed cooperative binding properties. To quantify this effect and to directly test the relative localization of YscQ_full_ and YscQ_C_, we expressed mCherry-YscQ_C_ *in trans* in an EGFP-YscQ_M218A_ background. Increasing levels of mCherry-YscQ_C_ led to a significant increase in the number of spots, rather than the intensity of spots, for both YscQ variants (Fig. 2C, 2D and 2F). Next, we analyzed the amount of mCherry-YscQ_C_ by immunoblot using polyclonal anti-YscQ antibodies (S4 Fig) and plotted the number of detected EGFP-YscQ_M218A_ spots against the amount of YscQ_C_ (Fig. 2E). The result shows a clear linear correlation (R^2^ > 0.98), suggesting that the formation of an observable focus is an all-or-nothing process and binding of full-size C-ring subunits to the injectisome is cooperative up to the maximal number of YscQ_full_ per injectisome. EGFP-YscQ_M218A_ and mCherry-YscQ_C_ foci colocalized (Fig. 2C). While YscQ_full_ required the presence of YscQ_C_ for its proper localization at the injectisome, overexpression of mCherry-YscQ_C_ did not remove EGFP-YscQ_M218A_ from its localization in foci at the injectisome (S5 Fig).

### The C-ring Component YscQ Exchanges between the Injectisome and the Cytosol

**Figure 3:**
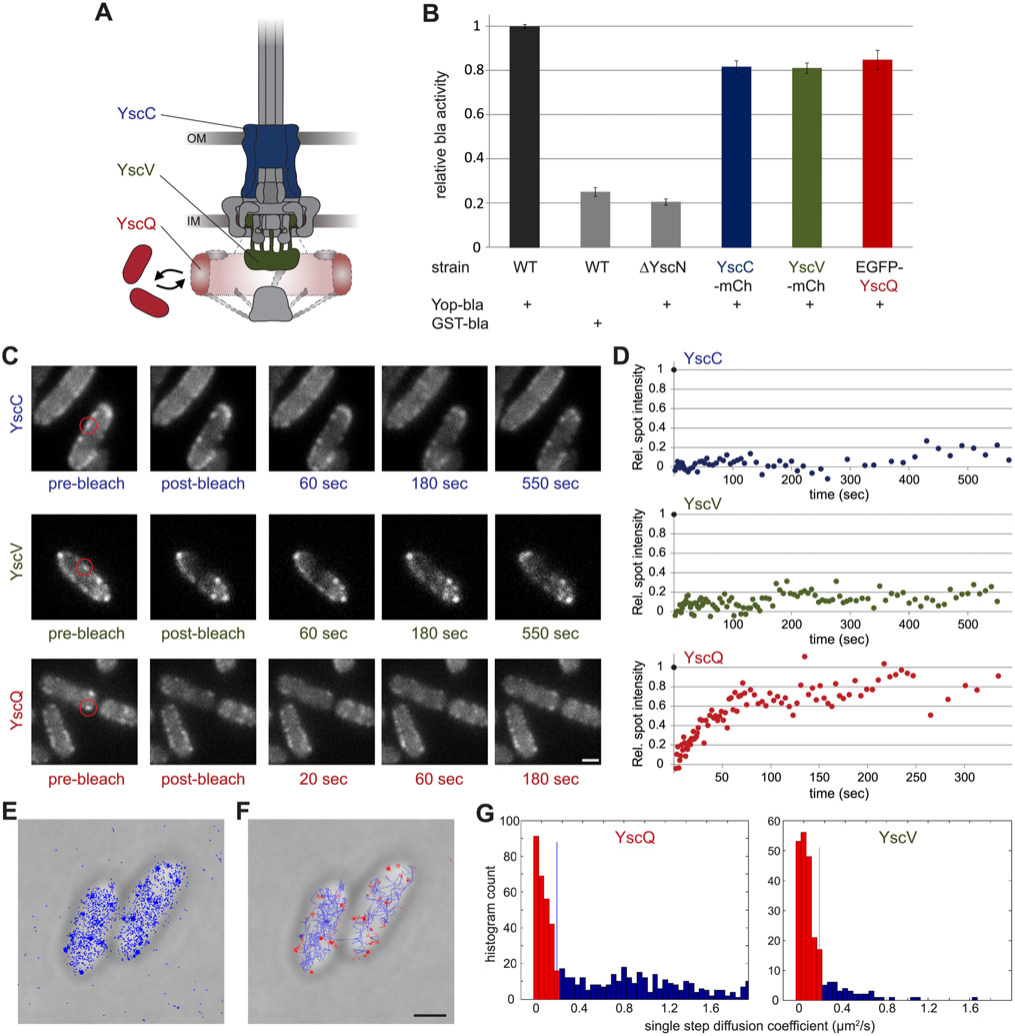
The C-ring subunit YscQ is a dynamic element of the injectisome. Fluorescent foci formed by EGFP-YscQ, but not by YscC-mCherry or YscV-mCherry show exchange with a cytosolic pool. (A) Position of the three studied components in the injectisome. Secretin YscC, blue; IM export apparatus component YscV, green; C-ring YscQ, red. Conformation and localization of the dashed components have not been experimentally determined. (B) Activity of strains expressing the indicated fusion proteins in an export kinetics assay based on the translocation of an artificial export substrate, YopH_1–17_-β-lactamase (Yop-bla). Wild-type control, set to 100%, black; negative controls (bla fused to the non-T3SS substrate glutathione-S-transferase GST and T3SS defective strain ΔYscN), grey. Error bars represent standard errors of the mean of *n* = 5 measurements (*n* = 4 for ΔYscN) in technical triplicates. (C) Micrographs showing representative images before and after photobleaching of a single fluorescent spot (marked by red circle) of YscC-mCherry, YscV-mCherry, and EGFP-YscQ. (D) Fluorescence recovery over time. Circles indicate the relative spot intensity in single frames for the micrographs shown in (C). (E) Localization of single PAmCherry-YscQ molecules in PALM. (F) Cellular distribution of motile (blue) and stationary (red) PAmCherry-YscQ molecules. (G) Single step diffusion coefficients for PAmCherry-YscQ and YscV-PAmCherry. See Materials and Methods for details. Scale bars, 1 μm. Numerical values and raw data (B, D, G) can be found in S1 Data.

Protein complexes can be dynamic assemblies, adapting their structure and function to changing environmental conditions. Given the inconsistent data on the cytosolic versus injectisome-bound localization of the C-ring, we wanted to test whether the C-ring exchanges subunits in the assembled structure. To this end, we followed the ability of injectisome components to exchange with cellular pools. Besides the C-ring subunit YscQ, we analyzed the secretin YscC and the major export apparatus component YscV, as these proteins cover the major functional subcomplexes of the injectisome (cytosolic components, membrane rings, and export apparatus, respectively) (Fig. 3A). All fusion proteins were stable and fully functional for secretion (S2 and S6 Figs.). To ensure that the fluorescent tags have only minimal influence on the kinetics of effector export, we devised a sensitive assay based on the quantification of export of an engineered T3SS substrate, β-lactamase fused to the YopH secretion signal, YopH_1–17_-bla [50–52]. In this assay, the strains expressing YscC-mCherry, YscV-mCherry, and EGFP-YscQ showed export rates of over 75% of the wild-type (WT) strain (Fig. 3B). This shows that the fluorescent tags have very little influence on protein functionality, suggesting that the exchange rates of labeled proteins resemble those of unlabeled subunits.

To detect the exchange of YscC-mCherry, YscV-mCherry, and EGFP-YscQ, we analyzed fluorescence recovery after photobleaching (FRAP) in live cells expressing these fusion proteins from their native promoters on the pYV virulence plasmid (Fig. 3C and 3D). Bacteria were grown and the T3SS was induced under non-secreting conditions. Three hours after induction, cells were transferred to secretion-permissive medium. A single fluorescent spot was photobleached by a short pulse of a tightly focused laser beam and recovery of fluorescence was analyzed for up to 10 minutes. As expected for the stable secretin ring, YscC-mCherry spots showed very little recovery within this time (Fig. 3C and 3D; S1 Movie). Similarly, YscV-mCherry spots only minimally recovered fluorescence (Fig. 3C and 3D; S2 Movie). In contrast, EGFP-YscQ spots showed almost complete recovery (≥75%) within the first few minutes after photobleaching (Fig. 3C and 3D; S3 Movie). Analysis of 20 recovery curves of EGFP-YscQ spots showed an average recovery half-time t_½_ of 68.2 ± 7.9 s. Similar to the full-length protein EGFP-YscQ, subunits of the C-terminal fragment EGFP-YscQ_C_ also exchanged between the injectisome and the cytosol (S7 Fig). While this exchange could not be quantified because N-terminally labeled YscQ_C_ can only be expressed *in trans*, this observation is compatible with exchange of a stable YscQ:YscQ_C_ complex. Individual recovery curves of EGFP-YscQ and EGFP-YscQ_C_ did not show any discernible large steps, which suggests that small units, such as single YscQ proteins or small subcomplexes, exchange within functional injectisomes.

To assess C-ring dynamics on a single-molecule level, we analyzed YscQ under secreting conditions by photoactivated localization microscopy (PALM) (Fig. 3E and 3F) [53–56]. We detected two populations of PAmCherry-YscQ molecules: some only moved within a very small area over successive exposures (Fig. 3F, red), whereas others were mobile (Fig. 3F, blue). Interestingly, the immobile PAmCherry-YscQ molecules were also tightly concentrated in small foci at the cell membrane (Fig. 3E and 3F). Analysis of the diffusion coefficient of PAmCherry-YscQ and YscV-PAmCherry (Figs. 3G and S8) showed that more than 50% of the PAmCherry-YscQ molecules were mobile (single step diffusion coefficients > 0.15 μm^2^/s), while only a minority of YscV-PAmCherry molecules were, confirming the different mobility of injectisome components on a single-molecule level.

### YscQ Exchange Is Linked to Effector Secretion and to the Function of the ATPase

**Figure 4:**
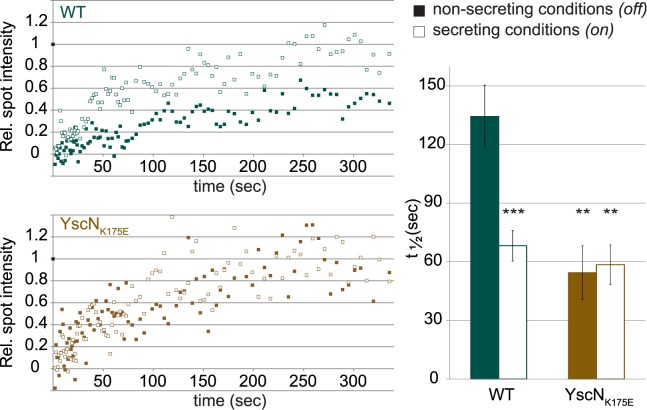
The C-ring is stabilized under non-secreting conditions in presence of the active ATPase. Representative recovery curves (left) and overall analysis of recovery half-times (right) of EGFP-YscQ in WT (turquoise) or YscN_K175E_ background (brown), under non-secreting conditions (full) or secreting conditions (open). Error bars represent the standard error of the mean from 19, 20, 9, and 17 data points (for WT non-secreting and secreting, YscN_K175E_ non-secreting and secreting, respectively). **, *p* < 0.01; ***, *p* < 0.001 compared to WT, non-secreting. The numerical values for the recovery half-times can be found in S1 Data.

To assess whether the exchange of YscQ between its docked location at the injectisome and the cytosolic pool might be functionally significant, we compared the rates of protein exchange under non-secreting and secreting conditions. We found that EGFP-YscQ was significantly less dynamic under non-secreting conditions, with an average half-time of recovery t_½_ of 134.3 ± 16.1 s, compared to a t_½_ under secreting conditions of 68.2 ± 7.9 s (Fig. 4). This result indicates that subunit exchange is regulated and links the observed turnover of YscQ to the ultimate function of the T3SS, effector secretion. Assembly of the C-ring depends on the presence of other cytosolic components, including the ATPase YscN [25]. However, the functional relation between the two components is unclear. Therefore, we analyzed YscQ dynamics in a strain expressing EGFP-YscQ and a catalytically inactive version of the ATPase, YscN_K175E_ [57]. Strains expressing YscN_K175E_ are defective in effector secretion [58], but form the C-ring [25]. We observed that the mutation in the ATPase led to fast EGFP-YscQ exchange both under non-secreting and secreting conditions, 54.4 ± 13.7 s and 58.5 ± 10.2 s, respectively (Fig. 4). The average mobile fraction was approximately 80% for both tested strains and conditions. These results show that the C-ring is only stabilized under non-secreting conditions when the ATPase is functional.

## Discussion

The role of the cytosolic components is a long standing open question in the type III secretion field. Despite the fact that all conserved cytosolic constituents are essential for assembly and function of the injectisome, their working mechanism and function in secretion remain unclear. This is especially true for the C-ring. Its main role in the closely related flagellum, the switching of rotation direction, does not apply to the injectisome, and its composition and behavior remained elusive.

The C-ring component SctQ has an internal translation initiation site leading to the expression of a protein equivalent to the C-terminal third of SctQ, SctQ_C_, in addition to the full-length protein, SctQ_full_ [32, 33]. Low levels of effector secretion of *Salmonella* SPI-2 lacking SctQ_C_ led to the proposal that the short version acts as a chaperone for SctQ_full_ [32]. The short translation product was however essential for secretion in *Y. pseudotuberculosis* [33]. In our present study, we found that the C-terminal fragment (YscQ_C_ in *Yersinia*) is required for the assembly of the cytosolic complex (Fig. 2). The requirement of YscQ_C_ for localization of YscQ_full_ and the colocalization of both proteins (Fig. 2C) is compatible with the proposal that binding of the two versions triggers structural rearrangements in YscQ_full_ [33]. Excess cytosolic YscQ_C_ did not titrate away YscQ_full_ from the injectisome (S5 Fig), showing that the affinity of YscQ_full_ to the complete localized cytosolic complex is higher than to YscQ_C_ alone. The loss of functionality in strains lacking the internal start site is therefore at least in part due to their inability to assemble the cytosolic components of the injectisome. This differs from the situation in the flagellum, where FliN (the homologue of YscQ_C_) stabilizes FliM (YscQ_full_), but is not absolutely required for correct FliM localization [59–61] and indicates divergent adaptation of the C-ring to its respective functions in the two systems.

The stoichiometry and size of the injectisome C-ring were unknown. We therefore compared the relative intensity of foci in strains expressing EGFP-YscQ or EGFP-YscD and, based on the well-documented reference SctD (YscD), which is present in 24 subunits [12, 41, 44–46], determined the number of full-length YscQ subunits per injectisome to be 22 both in non-secreting and secreting conditions (with standard deviations of 8 and 7, respectively) (Fig. 1). These numbers are based on an average occupancy and therefore represent a lower boundary for the maximum number of binding sites for a dynamic component. However, we did not detect a broader intensity distribution for YscQ compared to YscD (Fig. 1B and 1C), which would be expected if the number of YscQ per injectisome would fluctuate. As the stoichiometry of YscD is assumed to be constant, this suggests that despite the dynamic exchange of YscQ, the number of YscQ at the injectisome at any given time remains relatively constant and our numbers are close to the maximal stoichiometry. The absence of distinctive larger steps in the recovery curves after photobleaching in combination with the mutual requirement of YscQ_full_ and YscQ_C_ for assembly indicate that the (YscQ_full_):(YscQ_C_)_2_ complex proposed by Bzymek and colleagues [33] could be the building block of the injectisome C-ring.

In the *E. coli* and *Salmonella* flagellum, the C-ring is composed of about 36 FliM:FliN_4_ subcomplexes [61–63], but the C-rings from different species have diameters between 34 and 57 nm, suggesting the copy number might be different between species [64]. If the arrangement of YscQ_full_:YscQ_C_ subcomplexes within the machinery is similar to the flagellum, the diameter of the C-ring will be determined by the size and arrangement of YscQ_full_. Assuming a globular shape of YscQ_full_ and a circular arrangement of the subunits, the radius of a C-ring containing 22 YscQ_full_ is 30.2 nm (Fig. 1D; see S1 Text for details). The injectisome C-ring would therefore be smaller and composed of fewer subunits than its flagellar counterpart. Unlike the flagellar C-ring, the injectisome C-ring has not been visualized in recent cryotomography studies [41, 42], despite strong evidence that it is present at the cytosolic interface of the injectisome [20, 22, 25, 26]. The reason for this is unclear, but our observation that the injectisome C-ring is not fixed might at least partially explain this phenomenon. In a structural comparison of the flagellum and the injectisome, Kawamoto and colleagues [42] detected a weak electron density connecting the edge of the SctV “export gate” to an unknown and structurally indiscernible part at the cytoplasmic surface of the membrane. In the flagellum, a similar density connects the “export gate” FlhA with the C-ring. In the injectisome, the diameter of the binding site of the electron density at the cytoplasmic surface was approximately 28 nm [42], which is larger than the IM rings, but close to our calculated C-ring diameter of 30.2 nm. If the injectisome C-ring indeed resides at this spot, it would be located very close to the IM, which might further impede its structural identification.

Interestingly, we found that in functioning T3SSs, YscQ is not a stable structural component, but exchanges between a docking site at the injectisome and a cytosolic pool. The recovery half-time under secreting conditions, equivalent to the average lifetime of a given protein in the complex, was 68.2 ± 7.9 s. In contrast, the secretin SctC (YscC) and the major export apparatus component SctV (YscV) showed less than 20% recovery over 600 s (Fig. 3). This was expected in the case of the secretin, which forms a very stable ring in the outer membrane [65]. The flagellar homologue of SctV, FlhA, was shown to display some mobility [59], but in a more recent study, the majority of FlhA foci did not show any detectable turnover in the 10 minutes after photobleaching [66]. Our data show that similarly, there is little or no SctV turnover in the injectisome, in agreement with the proposition that the IM export apparatus is anchored relatively stable within the MS-ring [67].

Protein complexes can be highly dynamic assemblies, with proteins exchanging and switching their conformation while the complex is actively functioning. Dynamic behavior has been shown for the flagellum, where the stator protein MotB [68] and the C-ring component FliM exchange [63, 69–71]. Interestingly, FliM exchange was strongly decreased when clockwise rotation was suppressed, both in the absence of CheY [69] and when the flagellar motor was locked in counterclockwise direction [71]. We therefore tested whether the exchange of C-ring subunits in the injectisome is linked to the function of the T3SS, translocation of substrates. Comparing C-ring dynamics under secreting and non-secreting conditions, we found that the YscQ exchange rate was decreased under non-secreting conditions in the WT strain (Fig. 4). Which factors might control or influence this change of dynamics? The easiest explanation would be that the C-ring shuttles effector-chaperone complexes from the cytosol to the injectisome. However, such a direct role, similar to the one proposed for the flagellar ATPase complex [72] appears at odds with the high observed rates of effector export, once the injectisome is activated for secretion [73, 74]. Schlumberger and colleagues reported export rates of about 20 effector proteins per second [74], which are much faster than the observed exchange rates of the C-ring, although our study was performed in a strain lacking the six main effectors YopH, O, P, E, M, T, and both the presence of these effectors and direct cell contact as opposed to secretion induced by Ca^2+^ depletion might influence the exchange rate of the C-ring. Likewise, dynamics are not simply controlled by extracellular Ca^2+^ concentrations nor solely by the secretion process itself, as subunit exchange was fast regardless of extracellular Ca^2+^ in an ATPase-inactive strain (Fig. 4). Regulation of C-ring dynamics therefore differs from the situation in the flagellar C-ring, where exchange correlates with the output, the rotation direction, regardless of how this output was caused [63]. Taken together, the results suggest that YscQ is actively stabilized in the non-secreting mode and that this stabilization requires the ATPase to be functional. In these conditions, the ATPase is probably bound to one or more chaperone-substrate complexes. Combined with earlier observations that the C-ring itself binds to cargo [23, 26], these results suggest that the C-ring might be stabilized by the chaperone-substrate complexes bound to the ATPase under non-secreting conditions. The resulting close functional connection of C-ring and ATPase is supported by previous data showing that both proteins require each other for assembly [25]. Whether the dynamic exchange extends to the ATPase and the two other cytosolic components, SctK and SctL, which are all required to localize the C-ring, remains to be determined. At the moment, the lack of sufficiently active fusion proteins prevents the detailed analysis of these components. Based on structural arguments, Kawamoto and colleagues [42] proposed that in the flagellum, a structural rearrangement occurs between the “on” and “off” state, possibly involving the regulator protein (FliH in the flagellum, SctL in the injectisome), which in the flagellum was shown to be required to connect the ATPase FliI both to the C-ring (FliN) [75] and the major export apparatus component (FlhA) [76]. Such a structural rearrangement might explain our observation that C-ring dynamics decrease under non-secreting (=“off”) conditions. Interaction studies under different conditions might reveal the structural and functional rearrangements upon activation of export, an important step towards understanding the molecular mechanism of type III secretion.

Many bacteria utilize the T3SS to establish permanent infections and therefore need to adapt the function of the injectisome to their current environment. Within the host, regulating long-term activity of the system is critical for the survival of the bacterium, however this activity and its functional regulation have not been studied in detail. The steps in type III secretion that are governed by the cytosolic components, selection and export of substrates, are obvious targets for regulation and most signaling pathways employ cytosolic response regulators. Therefore, it is conceivable that the cytosolic components are involved in regulating type III secretion. Dynamic exchange is a suitable target for regulation, as exemplified by the flagellum and the nuclear pore complex [77]. Exchange of C-ring subunits could therefore be used to regulate T3SS function, in line with the observed changes in dynamics under different conditions. To date, a small number of external conditions influencing type III secretion beyond activation has been described, notably oxygenation [78, 79] and pH [80]. The involved pathways are species specific, suggesting that the respective signal receivers at the injectisome are less conserved than other components. Intriguingly, parts of the C-ring protein, especially its N-terminal region, show a significantly higher sequence variation than most other injectisome components (S9 Fig) [81], which could be an evolutionary consequence of responding to cues from species-specific signaling pathways.

Over the last years, understanding of the composition, function, and regulation of the functional cytosolic components of the T3SS has lagged behind the determination of the structure of the membrane rings. Our discovery that the C-ring is composed of around 22 subunits and requires the presence of additional copies of its C-terminal fragment to assemble sheds light on the composition of this essential part of the T3SS. Furthermore, our finding that the C-ring is a dynamic component and that its exchange correlates both with the secretion status and the activity of the ATPase reveals a new aspect of how the injectisome works and responds to its environment, advancing our knowledge and appreciation of the molecular mechanisms and regulation of the complete T3SS.

## Materials and Methods

### Bacterial Strains, Plasmids, and Genetic Constructions

Strains and constructs used in the experiments are listed in S2 Table. *E. coli* Top10 and BW19610 were used for cloning and *E. coli* SM10 λ pir^+^ for conjugation. *E. coli* strains were grown routinely on Luria–Bertani (LB) agar plates or in liquid LB medium at 37°C. Ampicillin and streptomycin were used at concentrations of 200 µg/ml and 100 µg/ml to select for expression vectors and suicide vectors. All *Y. enterocolitica* strains were based on strain IML421*asd* [41], which lacks the effectors YopH, YopO, YopP, YopE, YopM, and YopT and is auxotrophic for diaminopimelic acid due to an additional mutation in the aspartate-β-semialdehyde *(asd)* gene. *Y. enterocolitica* were routinely grown at 25°C in brain heart infusion (BHI) broth containing nalidixic acid (35 µg/ml) and diaminopimelic acid (80 µg/ml). Plasmids were generated using Phusion polymerase (Finnzymes). Mutators for modification or deletion of genes in the pYV plasmids were constructed as described earlier [49]. All constructs were confirmed by sequencing (Source BioScience).

*Y. enterocolitica* mutants were generated by allelic exchange, replacing the WT gene on the virulence plasmid by the mutated version. Completion of the allelic exchange was tested for by plating diploid bacteria on plates containing 5% sucrose [82].

### *Y. enterocolitica* Culture Conditions

*Y. enterocolitica* cultures for secretion assays and stoichiometry analysis were inoculated from stationary overnight cultures to an optical density at 600 nm (OD_600_) of 0.12 in BHI broth containing EDTA (5 mM) (BHI-EDTA, secreting conditions) or CaCl_2_ (5 mM) (BHI-Ca^2+^, non-secreting conditions) supplemented with nalidixic acid (35 µg/ml), diaminopimelic acid (80 µg/ml), glycerol (4 mg/ml), and MgCl_2_ (20 mM). After 1.5 h of growth at 25°C, the *yop* regulon was induced by shifting the culture to 37°C. Where indicated, expression of the pBAD constructs was induced by adding L-arabinose (0.2%, unless stated otherwise) to the culture just before the shift to 37°C. After 3 h of incubation at 37°C, cultures were used for further analysis.

At this point, bacteria for FRAP and PALM analysis were collected (2,400*g*, 4 min) and resuspended in HEPES-M22 complemented with diaminopimelic acid (80 µg/ml) and either 5 mM EDTA (secreting conditions) or 5 mM CaCl_2_ (non-secreting conditions).

### Secretion Analysis and Immunoblotting

Bacteria and supernatant (SN) fractions were separated by centrifugation at 20,800*g* for 10 min at 4°C. The cell pellet was taken as total cell (TC) fraction. Proteins in the SN were precipitated with trichloroacetic acid 10% (w/v) final for 1–8 hours at 4°C. Proteins were separated on Novex 4%–20% gradient SDS-PAGE gels (Life technologies). Unless mentioned otherwise, proteins secreted by 3 × 10^8^ bacteria (SN) or produced by 2.5 × 10^8^ bacteria (TC) were loaded per lane. Secreted proteins were stained using the Coomassie-based “Instant blue” staining solution (Expedeon). Immunoblotting was carried out using mouse polyclonal antibodies against GFP (Clontech 632459, 1:1,000) or mCherry (Clontech 632543, 1:1,000) or rabbit polyclonal antibodies against YscQ (MIPA235, 1:1,000). Detection was performed with corresponding rabbit anti-mouse or goat anti-rabbit secondary antibodies conjugated to horseradish peroxidase (Dako; 1:5,000), before development with Immobilon Western chemiluminescent substrate (Millipore). For the quantification of YscQ_C_, the signal intensity of the region around the expected protein size in each lane of the immunoblot was measured using ImageJ and corrected for the background intensity in the lane lacking YscQ_C_.

### β-lactamase Assay

*Y. enterocolitica* strains were transformed with plasmids pAD372 or pAD374 (expressing GST-bla or YopH_1–17_-bla under an arabinose-inducible promoter) (see S2 Table). Cultures were inoculated and grown under non-secreting conditions as stated above. Expression was induced with 0.2% Arabinose (w/v) in parallel to the temperature switch to 37°C. Bacteria were collected by centrifugation (4 min, 2,400*g*, 37°C), and resuspended in prewarmed HEPES-M22 (M22 buffered with 20 mM HEPES instead of phosphate buffer) complemented with diaminopimelic acid (80 µg/ml), glycerol (4 mg/ml), and MgCl_2_ (20 mM) and 5 mM EDTA to induce secretion, followed by 30 min incubation at 37°C. In a 96 well Corning 3603 plate, 100 µl/well of each strain was added in triplicates and 20 µl/well of β-lactamase substrate solution (0.1 M Tris-HCl [pH 7.5], 20 µM Fluoricillin Green 495/525 [Life Technologies]) was added. Fluorescence was measured every 30 s for 15 min, using a BMG Labtech Fluostar photometer (Ex 490/10 nm, Em 530/12 nm) and the slope of the linear increasing region was determined. The results are averages of five independent experiments (four independent experiments for strain ΔYscN) with three technical replicates each.

### Fluorescence Microscopy

For standard fluorescence imaging, determination of spot intensity, and deconvolution, 1.5 µl of resuspended bacterial culture was placed on a microscope slide layered with a pad of 2% agarose in HEPES-M22 (M22 buffered with 20 mM HEPES instead of phosphate) supplemented with diaminopimelic acid (80 µg/ml) and either EDTA (5 mM) (secreting conditions) or CaCl_2_ (5 mM) (non-secreting conditions). A Deltavision Spectris optical sectioning microscope (Applied Precision) equipped with a UPlanSApo 100 × 1.40 oil objective (Olympus) combined with 1.6× auxiliary magnification and an Evolve EMCCD camera (Photometrics) was used to take differential interference contrast (DIC) and fluorescence photomicrographs. For fluorophore visualization, either the GFP/hsGFP filter set (Ex 475/28 nm, Em 522/44 nm) or the mCherry/hsCherry filter set (Ex 575/25 nm, Em 634/63 nm) were used. DIC frames were taken with 0.05 s and fluorescence frames with 1.0 s exposure time. Per image, a Z-stack containing 7 to 15 frames per wavelength with a spacing of 150 nm was acquired. The stacks were deconvolved using softWoRx 5.5 with standard settings (Applied Precision). A representative DIC frame and the corresponding fluorescence frame were selected and further processed with the ImageJ software. Quantitative analysis was performed on the undeconvolved images.

### Fluorescence Recovery after Photobleaching

For FRAP, a custom-built multi-color fluorescence microscope was used, which allows photobleaching of one fluorescent spot within the cell using slimfield while simultaneously monitoring the fluorescence emission using conventional widefield epifluorescence [68, 83]. 1.5 µl of resuspended bacterial culture were analyzed on a microscope slide layered with a pad of 2% agarose in the respective resuspension medium. Fluorescence was excited using a 473 nm or a 561 nm laser (for EGFP or mCherry, respectively). Fluorescence emission was imaged at 50 nm∕pixel in frame-transfer mode at 25 Hz by a 128 × 128-pixel, cooled, back-thinned electron-multiplying charge-coupled device camera (iXon DV860-BI; Andor Technology). The displayed images are unweighted averages of three consecutive frames. Analysis was performed on the raw data. Average fluorescence intensities of the spot (I_S_), the corresponding bacterium (I_B_), and the background (I_0_) were determined manually using ImageJ. The spot intensity ratio R = (I_S_ − I_0_) / (I_B_ − I_0_) was calculated for each frame. The pre-bleach ratio and post-bleach ratio were determined using ten frames immediately before and after photobleaching the spot with a 20 ms focused laser beam. Unless stated otherwise, the recovery curve was measured by 100 frames of 40 ms exposure over a period of 335–570 s. Values were normalized with the pre-bleach and post-bleach ratio and τ_½_ was calculated by fitting R(time) to an exponential curve using the formula R(time) = start + (start-end) * (Exp(−time/τ_½_) − 1) using the sequential quadratic programming algorithm in SPSS Statistics 21 (IBM), with the parameters set to start = [0.9; 1.1], end = [−0.1; 0.4]. Fits with an r^2^ value of less than 0.4 were excluded from further analysis. The recovery half-time (or half-life) t_½_ was calculated using he formula t_½_ = τ_½_* ln(2).

**Photoactivated Localization Microscopy** *Y. enterocolitica* cultures were grown and treated as given above for FRAP analysis. 15,000 consecutive frames were imaged with a frame rate of 15.26 ms on a custom-built single-molecule fluorescence microscope using a 405-nm laser (CNI) for photoactivation and a 561-nm laser (Oxxius) for PAmCherry excitation. Analysis was performed using the STORMTRACKER package for MATLAB (MathWorks) as described by Uphoff and colleagues [53]. The minimal track length was 5, the threshold used to discriminate between bound and unbound molecules was 0.15 μm^2^/s for the single-step diffusion coefficient.

### Spot Detection and Determination of Intensities

The stoichiometry of YscQ was determined comparing the fluorescence intensities of fluorescent foci in strains expressing EGFP-YscQ or EGFP-YscD under secreting and non-secreting conditions. Both proteins were expressed from their native promoter on the pYV virulence plasmid. Images were corrected for background fluorescence only and were not processed otherwise to allow for direct comparison of fluorescence distribution and intensity. Automated spot counting in fluorescent 3D stacks was performed in MATLAB (MathWorks) using the electron tomography related Dynamo toolbox (http://dynamo-em.org/) [84]. The peaks were detected based on the fluorescent intensity being higher than the average background of the stack by 5 standard deviations of the pixel values. The minimal distance between two peaks was limited by 3 pixels (304 nm). For each strain and condition, 11 stacks from two independent experiments were imaged. In each stack, at least 100 spots were detected, the total number of analyzed spots was between 3,400 and 4,300 per condition. In parallel, ten stacks from two independent experiments were imaged for cells not expressing any fluorophore. Spot intensity was corrected for the background outside of cells and the average fluorescence background in cells not expressing a fluorophore. Each stack was treated as an independent value. Spot detection and quantification for EGFP-YscQ_M218A_ at different mCherry-YscQ_C_ levels was performed using MicrobeTracker 0.937 [85] and the automated SpotFinderZ algorithm using the following settings: Expand cells, 2 px; max width squared, 22.3 px^2^; min width squared, 1.11 px^2^; min height, 0.053 i.u.; max rel. sq. error, 97; max var/sq. height ratio, 1.23; min filtered/fitted ratio, 0.02187; and standard settings otherwise. More than 170 cells per condition were analyzed.

The numerical data used in all figures (Figs. 1–4) are included in S1 Data.

## Supporting Information

S1 Data Numerical values and raw data. Numerical values and raw data used to generate Figs. 1B, 1C, 2D, 2E, 2F, 3B, 3D, 3G, and 4.
(XLSX)

S1 Fig Schematic representation of the type III secretion injectisome. Conformation and localization of the dashed components (names in italics) have not been experimentally determined. Protein names in black: general Sct nomenclature [3]; brown: *Yersinia* nomenclature.
(EPS)

S2 Fig Stability and functionality of the EGFP fusion proteins used in this study. (A) Immunoblot anti-GFP of total cellular proteins in a wild-type strain and strains expressing EGFP-YscD or EGFP-YscQ from their native promoter on the pYV virulence plasmid, or EGFP-YscQ_C_ *in trans* in a YscQ_M218A_ background. To assess the stability of the fusion proteins, untreated total cellular proteins were analyzed using anti-GFP antibodies. All samples were run on the same gel, the grey lines denote the omission of intermediate lanes. (B) Secretion assay showing the secreted proteins in the strains shown in (A), as well as the negative control ΔYscN and YscQ_M218A_ with no plasmid or expressing YscQ_C_ *in trans*. (C) Secretion assay showing the Calcium regulation of tested strains. Translocated proteins under secreting conditions (absence of calcium) in the WT strain (left side, black) and under non-secreting conditions (presence of calcium) in the strains shown in the strains shown in (B) (right side, green).
(EPS)

S3 Fig YscQ_M218A_, YscQ_C_, and YscN require each other for localization at the injectisome. (A) Fluorescence micrographs showing the distribution of EGFP-YscQ_C_ expressed *in trans* in strains expressing YscQ_M218A_ (left) or no YscQ (right). (B) Distribution of EGFP-YscN expressed *in trans* in strains lacking native YscN and otherwise wild-type (left) or YscQ_M218A_ (right). Insets show enlarged parts of the main frames.
(EPS)

S4 Fig Determination of mCherry-YscQ_C_ protein levels at different induction levels. The amount of mCherry-YscQ_C_ in a strain expressing EGFP-YscQ_M218A_ (from its native promoter on the virulence plasmid) and mCherry-YscQ_C_ (not expressed in the left lane and expressed *in trans* by induction of the plasmid with the given concentrations of arabinose in the other lanes) was determined by an immunoblot anti-YscQ of total cellular proteins. Amount of detected YscQ_C_ per condition (in arbitrary units, left lane = no YscQ_C_ set to 0), from left to right: 0.00, 1.59, 2.4, 4.41, 5.12, 9.93, 20.73.
(EPS)

S5 Fig Overexpression of YscQ_C_ does not titrate YscQ_M218A_ away from the injectisome. Cellular distribution of EGFP-YscQ_M218A_ (expressed from its native promoter on the virulence plasmid) and mCherry-YscQ_C_ (overexpressed *in trans* induced by 0.08% arabinose). The increased cytosolic level of YscQ_C_ does not interfere with the localization of EGFP-YscQ_M218A_ at the injectisome.
(EPS)

S6 Fig Stability and functionality of the YscV-mCherry and YscC-mCherry fusion proteins. (A) Immunoblot anti-mCherry of total cellular proteins in a wild-type strain and strains expressing YscV-mCherry or YscC-mCherry from their native promoters on the pYV virulence plasmid. To assess the stability of the fusion proteins, untreated total cellular proteins were analyzed using anti-mCherry antibodies. YscV-mCherry and YscC-mCherry run at single bands at the expected size of the fusion proteins (indicated at the bottom). (B) Secretion assay showing the secreted proteins in the strains used in (A). (C) Secretion assay showing the calcium regulation of tested strains. Translocated proteins under secreting conditions (absence of calcium) in the WT strain (left side, black) and under non-secreting conditions (presence of calcium) in the strains shown in (B) (right side, green).
(EPS)

S7 Fig EGFP-YscQ_C_ subunits exchange in the active injectisome in a similar manner as EGFP-YscQ. Micrographs showing representative images before and after photobleaching of a single fluorescent spot (marked by red circle). While recovery of EGFP-YscQ_C_ was generally observable, the expression of the analyzed protein *in trans* lead to cell-to-cell variations of YscQ_C_ protein levels and higher background fluorescence, preventing the quantification of the exchange rate.
(EPS)

S8 Fig Functionality of the PAmCherry fusion proteins used in this study. (A) Secretion assay showing the secreted proteins in a wild-type strain, a strain lacking the ATPase YscN and strains expressing PAmCherry-YscQ or YscV-PAmCherry from their native promoters on the pYV virulence plasmid. All samples were run on the same gel, the grey line denotes the omission of intermediate lanes. (B) Secretion assay showing the Calcium regulation of tested strains. Translocated proteins under secreting conditions (absence of Calcium) in the WT strain (left side, black) and under non-secreting conditions (presence of Calcium) in a WT strain and strains expressing PAmCherry-YscQ or YscV-PAmCherry from their native promoters on the pYV virulence plasmid (right side, green).
(EPS)

S9 Fig Conservation heat map of the type III secretion system. (A) The degree of sequence conservation of several subunits was determined using the similarity score of the consensus sequence determined by the multiple sequence alignment package M-Coffee [86] for representatives of major T3SS subfamilies [81]. More conserved subunits are represented by darker colors. Proteins with very low molecular weight or low number of known homologues are displayed in grey. Conformation and localization of the dashed components have not been experimentally determined. (B) Multiple sequence alignment of SctQ proteins from different T3SS, top to bottom: *Y. enterocolitica* YscQ (AAD16827), *Ralstonia solanacearum* HrcQ (CAD18012), *Pseudomonas syringae* HrcQ_A+B_ (AAO54919, AA054918), *E. coli* SepQ (CAS11493), *Salmonella* Typhimurium SPI-1 SpaO (CBW18968). A plain text alignment can be accessed in S2 Text.
(EPS)

S1 Movie Fluorescence recovery after photobleaching of YscC-mCherry. To reduce background levels, the displayed frames are walking averages of three 40 ms exposed frames each (except the bleach frame). After photobleaching with a 20 ms frame of a tightly focused laser beam, the frame rates were: 10 × 2 s, 10 × 4 s, 10 × 6 s, 10 × 10 s, 10 × 15 s, 10 × 20 s.
(AVI)

S2 Movie Fluorescence recovery after photobleaching of YscV-mCherry. As S1 Movie, except for the following frame rates after photobleaching: 25 × 2 s, 25 × 4 s, 25 × 6 s, 25 × 10 s.
(AVI)

S3 Movie Fluorescence recovery after photobleaching of EGFP-YscQ. As S1 Movie, except for the following frame rates after photobleaching: 25 × 1 s, 25 × 2 s, 25 × 4 s, 26 × 6 s.
(AVI)

S1 Table Homologous proteins and their function in various families of T3SS and the flagellum. General Sct names [3] and protein names are given for well-studied members of the respective T3SS families. -, no clear homologue present.
(DOCX)

S2 Table List of strains and plasmids used in this study. Relevant genotype of *Y. enterocolitica* strains. All strains are based on the multi-effector knock-out auxotrophic strain IML421*asd*, which is an E40-based strain.
(DOCX)

S1 Text Calculation of the C-ring diameter. (DOCX)

S2 Text Plain text alignment of the sequences compared in S9B Fig. (TXT)

## Abbreviations

FRAP: fluorescence recovery after photobleaching
IM: inner membrane
PALM: photoactivated localization microscopy
pYV: plasmid for *Yersinia* virulence
T3SS: type III secretion system
WT: wild-type
