# Supplementary material for: Composition, Formation, and Regulation of the Cytosolic C-ring, a Dynamic Component of the Type III Secretion Injectisome
Source: PLoS Biol. 2015 Jan 15;13(1):e1002039. doi: 10.1371/journal.pbio.1002039 (PMC4295842; doi:10.1371/journal.pbio.1002039)
Supplement: S1 Table — General Sct names [3] and protein names are given for well-studied members of the respective T3SS families. -, no clear homologue present. (DOCX) [file pbio.1002039.s014.docx]

**Table S1**

**Homologous proteins and their function in various families of T3SS and the flagellum**

General Sct names [1] and protein names are given for well-studied members of the respective T3SS families. -, no clear homologue present.

| **Functional name** | **Sct name** | ***Yersinia*** | ***Shigella*** | ***Salmonella SPI-1*** | ***Escherichia coli*** | **Flagellar homologue** |
| --- | --- | --- | --- | --- | --- | --- |
| Secretin | SctC | YscC | MxiD | InvG | EscC | - |
| Outer MS-ring protein | SctD | YscD | MxiG | PrgH | EscD | - |
| Inner MS-ring protein | SctJ | YscJ | MxiJ | PrgK | EscJ | FliF |
| Minor export apparatus protein | SctR | YscR | Spa24 (SpaP) | SpaP | EscR | FliP |
| Minor export apparatus protein | SctS | YscS | Spa9 (SpaQ) | SpaQ | EscS | FliQ |
| Minor export apparatus protein | SctT | YscT | Spa29 (SpaR) | SpaR | EscT | FliR |
| Export apparatus switch protein | SctU | YscU | Spa40 (SpaS) | SpaS | EscU | FlhB |
| Major export apparatus protein | SctV | YscV | MxiA | InvA | EscV | FlhA |
| Accessory cytosolic protein | SctK | YscK | MxiK | OrgA | - | - |
| C-ring protein | SctQ | YscQ | Spa33 (SpaO) | SpaO | EscQ | FliM + FliN |
| Regulator (stator) | SctL | YscL | MxiN | OrgB | EscL (Orf5) | FliH |
| ATPase | SctN | YscN | Spa47 (SpaL) | InvC | EscN | FliI |
| Stalk | SctO | YscO | Spa13 (SpaM) | InvI | Orf15 | FliJ |
| Needle filament protein | SctF | YscF | MxiH | PrgI | EscF | - |
| Inner rod protein | SctI | YscI | MxiI | PrgJ | EscI (rOrf8) | - |
| Needle length regulator | SctP | YscP | Spa32 (SpaN) | InvJ | EscP (Orf16) | FliK |
| Hydrophilic translocator, needle tip protein |  | LcrV | IpaD | SipD | - | - |
| Hydrophobic translocator, pore protein |  | YopB | IpaB | SipB | EspD | - |
| Hydrophobic translocator, pore protein |  | YopD | IpaC | SipC | EspB | - |
| Pilotin |  | YscW | MxiM | InvH | - | - |
| Gatekeeper | SctW | YopN | MxiC | InvE | SepL | - |

Reference:

1. Hueck CJ (1998) Type III protein secretion systems in bacterial pathogens of animals and plants. Microbiol Mol Biol Rev 62: 379–433.
