# Supplementary material for: Composition, Formation, and Regulation of the Cytosolic C-ring, a Dynamic Component of the Type III Secretion Injectisome
Source: PLoS Biol. 2015 Jan 15;13(1):e1002039. doi: 10.1371/journal.pbio.1002039 (PMC4295842; doi:10.1371/journal.pbio.1002039)
Supplement: S2 Table — Relevant genotype of Y. enterocolitica strains. All strains are based on the multi-effector knock-out auxotrophic strain IML421asd, which is an E40-based strain. (DOCX) [file pbio.1002039.s015.docx]

**S2 Table**

**List of strains and plasmids used in this study**

Relevant genotype of *Y. enterocolitica* strains. All strains are based on the multi-effector knock-out auxotrophic strain IML421*asd*, which is an E40-based strain.

| Strain name | Relevant strain characteristics | References |
| --- | --- | --- |
| IML421 | E40, pYVe40 (*yopO_Δ2-427_ yopE_21_ yopH_Δ1-352_ yopM_23_ yopP_23_ yopT_135_*) | [1] |
| IML421*asd* | E40, pYVe40 (*yopO_Δ2-427_ yopE_21_ yopH_Δ1-352_ yopM_23_ yopP_23_ yopT_135_*) *Δasd* | [2] |
| AD4085 | IML421*asd* *egfp-yscQ* | [2] |
| AD4165 | IML421*asd* *ΔyscN (mutated with pAD168)* | this work |
| AD4167 | IML421*asd*  *yscC-mCherry (mutated with pMA12)* | this work |
| AD4306 | IML421*asd* *egfp-yscD (mutated with pAD306)* | this work |
| AD4334 | IML421*asd* *yscV-mCherry (mutated with pAD334)* | this work |
| AD4350 | IML421*asd* *yscQ_M218A_ (mutated with pAD350)* | this work |
| AD4396 | IML421*asd* *egfp-yscQ_M218A_ (mutated with pAD356)* | this work |
| AD4419 | IML421*asd* *ΔyscQ (mutated with pAD419)* | this work |
| AD4422 | IML421*asd* *egfp-yscQ yscN_K175E_   (AD4085 mutated with pAD420)* | this work |
| AD4425 | IML421*asd* *yscQ_M218A_* *ΔyscN  (AD4350 mutated with pAD168)* | this work |
| AD4441 | IML421*asd* *yscV-pamCherry1 (mutated with pAD441)* | this work |
| AD4443 | IML421*asd* *pamCherry1-yscQ (mutated with pAD443)* | this work |

Expression and suicide vectors

| Plasmid | Relevant characteristics | References |
| --- | --- | --- |
| pAD143 | pBAD::*egfp-yscQ* (complete *yscQ* gene with N-terminal *egfp*) | this work |
| pAD168 | pKNG101 *ΔyscN* | [3] |
| pAD306 | pKNG101 *egfp-yscD* (*egfp* and flexible linker cloned in-frame at the N-terminus of *yscD*) | this work |
| pAD334 | pKNG101 *yscV-mCherry* (*mcherry* and flexible linker cloned in-frame at the C-terminus of *yscV*) | this work |
| pAD341 | pBAD::*egfp-yscN (complete yscN gene with N-terminal egfp)* | this work |
| pAD350 | pKNG101 *yscQ_M218A_* (*mutator introducing Met to Ala substitution at position 218 of yscQ*) | this work |
| pAD351 | pBAD::*yscQ_C_ (C-terminal fragment of yscQ corresponding to the product of the internal translation start at Met-218)* | this work |
| pAD356 | pBAD::*egfp-yscQ_C_ (egfp and flexible linker cloned in-frame with C-terminal fragment of yscQ from Met-218)* | this work |
| pAD359 | pBAD::*mCherry-yscQ_C_ (mcherry and flexible linker cloned in-frame with C-terminal fragment of yscQ from Met-218)* | this work |
| pAD372 | pBAD::*gst-bla (β-lactamase from pBAD::His-B cloned in-frame with glutathion S-transferase from pGEX-6P-1)* | this work |
| pAD374 | pBAD::*yopH_1-17_-bla (β-lactamase from pBAD::His-B cloned in-frame with first 17 amino acids of Y. enterocolitica yopH)* | this work |
| pAD419 | pKNG101 *ΔyscQ* | this work |
| pAD420 | pKNG101 *yscN_K175E_* (*mutator introducing Lys to Glu substitution at position 218 of yscQ*) | this work |
| pAD441 | pKNG101 *yscV-pamCherry* (*pamCherry* and flexible linker cloned in-frame at the C-terminus of *yscV*) | this work |
| pAD443 | pKNG101 *pamCherry-yscQ* (*pamCherry* and flexible linker cloned in-frame at the N-terminus of *yscQ*) | this work |
| pBAD::*his*/B | pBR322-derived expression vector | Invitrogen |
| pKNG101 | ori_R6K_ *sacBR^+^* oriT_RK2_ *strAB^+^* (suicide vector) | [4] |
| pMA12 | pKNG101 *yscC-mCherry*^+^ (*mCherry* cloned in-frame at the C-terminus of *yscC*) | [5] |

References

1. Iriarte M, Cornelis GR (1998) YopT, a new Yersinia Yop effector protein, affects the cytoskeleton of host cells. Mol Microbiol 29: 915–929. doi:10.1046/j.1365-2958.1998.00992.x.

2. Kudryashev M, Stenta M, Schmelz S, Amstutz M, Wiesand U, et al. (2013) In situ structural analysis of the Yersinia enterocolitica injectisome. Elife 2: e00792. doi:10.7554/eLife.00792.

3. Diepold A, Wiesand U, Cornelis GR (2011) The assembly of the export apparatus (YscR,S,T,U,V) of the Yersinia type III secretion apparatus occurs independently of other structural components and involves the formation of an YscV oligomer. Mol Microbiol 82: 502–514. doi:10.1111/j.1365-2958.2011.07830.x.

4. Kaniga K, Delor I, Cornelis GR (1991) A wide-host-range suicide vector for improving reverse genetics in gram-negative bacteria: inactivation of the blaA gene of Yersinia enterocolitica. Gene 109: 137–141.

5. Diepold A, Amstutz M, Abel S, Sorg I, Jenal U, et al. (2010) Deciphering the assembly of the Yersinia type III secretion injectisome. EMBO J 29: 1928–1940. doi:10.1038/emboj.2010.84.
