# Supplementary material for: Composition, Formation, and Regulation of the Cytosolic C-ring, a Dynamic Component of the Type III Secretion Injectisome
Source: PLoS Biol. 2015 Jan 15;13(1):e1002039. doi: 10.1371/journal.pbio.1002039 (PMC4295842; doi:10.1371/journal.pbio.1002039)
Supplement: S1 Text — (DOCX) [file pbio.1002039.s016.docx]

**S1 Text**

**Calculation of the C-ring diameter**

Molecular weight of YscQ_full_ = 34,412.6 Da (Gen Bank accession AAD16827).

Assuming an average partial specific volume v_2_ = 0.73 cm^3^/g [1], the minimal radius of spherical protein is: R_min_ = (3V/4π)^1/3^ = 0.066 MW ^1/3^ (MW, molecular weight in Dalton: R_min_, minimal radius in nanometer) [1].

**R_min_(YscQ_full_)** = 0.066 * 34,412^1/3^ nm = **2.147 nm**

In a circle composed of 22 subunits, the radius of a single subunit corresponds to an angle of
α = 360/44 = 8.18 degrees.

**r(C-ring)** = R_min_(YscQ_full_) / sin(α) **= 15.1 nm**

Reference

1. Erickson HP (2009) Size and shape of protein molecules at the nanometer level determined by sedimentation, gel filtration, and electron microscopy. Biol Proced Online 11: 32–51. doi:10.1007/s12575-009-9008-x.
